# Genome Sequence of *Cotton Leafroll Dwarf Virus* Infecting Cotton in Georgia, USA

**DOI:** 10.1128/MRA.00812-20

**Published:** 2020-08-20

**Authors:** Afsha Tabassum, Phillip M. Roberts, Sudeep Bag

**Affiliations:** a Department of Plant Pathology, University of Georgia, Tifton, Georgia, USA; b Department of Entomology, University of Georgia, Tifton, Georgia, USA; Portland State University

## Abstract

Cotton leafroll dwarf disease (CLRDD), caused by the aphid-borne *Cotton leafroll dwarf virus* (CLRDV; genus, *Polerovirus*; family, *Luteoviridae*), has been recently reported from the major cotton-growing regions of the United States. Here, we present the nearly complete genome sequence of a CLRDV isolate from cotton in Georgia.

## ANNOUNCEMENT

**FIG 1:**
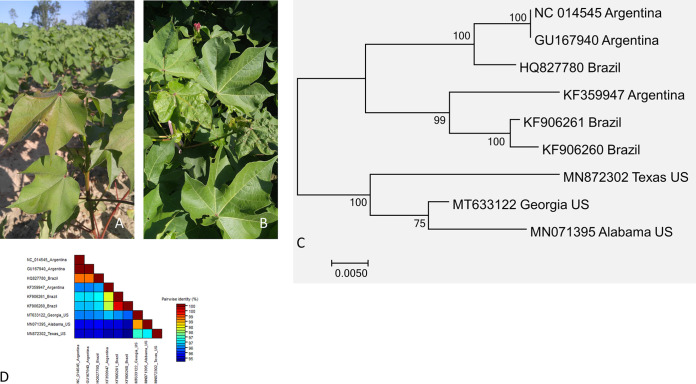
Symptomatology and sequence analysis of *Cotton leafroll dwarf virus* (CLRDV). Cotton plant with symptoms of reddening and drooling (A) and crinkling and deformation (B) of leaves. Maximum likelihood phylogenetic tree (C) and pairwise identity matrix (D) of nearly complete CLRDV nucleotide sequence from Georgia with other reported sequences from GenBank.

Cotton is the second most important agricultural commodity for the state of Georgia, with a farm gate value of $901.5 million (1). *Cotton leafroll dwarf virus* (CLRDV), a phloem-limited virus, is associated with the emerging cotton leafroll dwarf disease (CLRDD) in the United States. It was first reported from Alabama in 2019 (2) and subsequently from the major cotton-growing regions in the United States, including Florida (3), Georgia (4), Louisiana (5), Mississippi (6), South Carolina (7), and Texas (8). Symptoms of the disease include reddening of the leaves and petioles and drooling, crinkling, and deformation of the leaves (Fig. 1A and B), and it has the potential to cause significant yield and economic losses. The viral genome consists of a single-stranded positive-sense RNA approximately 5.8 kb long encoding seven different proteins (9, 10).

In summer 2018, symptomatic plants (*n* = 20) showing reddening, drooling of leaves, and reddening of petioles, along with asymptomatic plants (*n* = 20), were collected from three counties, Early, Seminole, and Tift, in Georgia. CLRDV was detected from symptomatic samples but not from the asymptomatic tissue tested (4). To understand the genomic composition of CLRDV from Georgia, a nearly complete genome of an isolate from Seminole County was sequenced using Sanger’s method and analyzed.

Total RNA was extracted from a pooled sample of symptomatic leaves, petioles, and bark tissues using the modified cetyltrimethylammonium bromide method (11, 12). Complementary DNA (cDNA) was synthesized from 2.5 μg of total RNA using Superscript III reverse transcriptase (Invitrogen, USA) and specific reverse primers targeting different open reading frames (ORFs) (Table 1) of the virus genome following the manufacturer’s recommended conditions. The cDNA (2 μl) and specific primer combinations (Table 1) were used to amplify different ORFs of the CLRDV genome using Platinum *Taq* DNA polymerase (Invitrogen, USA). Products of predicted sizes were cloned into the pGEM-T Easy I cloning vector (Promega, USA), and both strands were sequenced using the SP_6_ to T_7_ sequencing primers (GenScript, USA). The nearly complete nucleotide sequence was assembled from the consensus sequence of three clones for the target region. The sequence was annotated with the help of BioEdit (13) and MEGA X (14) software and submitted to GenBank (accession number MT633122). The maximum likelihood phylogenetic tree of nearly full-length nucleotide sequences was constructed using the CLRDV sequences from GenBank and the isolate sequenced in this study with MEGA X (14) software. Pairwise comparisons of the nucleotide sequences were performed with SDT v.2.1 (15) software.

**TABLE 1:** Sequences of oligonucleotide primer combinations, target regions, and annealing temperatures used to amplify the *Cotton leafroll dwarf virus* genome sequence in this study

Strain name	Sequence (5′–3′)	Primer position	Amplicon length (bp)	*T_m_*^*a*^ (°C)
SB19F	ACAAAAGAACGATAGAGGGGTTGTT	1–25	1,352	60
SB20R	ACCACCAACGTGGACTCCGAC	1352–1332		
SB28F	TTCAGGTGGATACAGTGGGAC	1286–1306	1,547	58
SB19R	GTGGGAACCAGGTATTCCCGC	2813–2833		
SB25F	CGCCTCATCATGTCTGTATCCC	2609–2630	1,075	56
SB15R	CCTACGTGGTCGTCTTCTTCCATTG	3683–3659		
SB11F	AGGTTTTCTGGTAGCAGTACCAATATCAACGTTA	3544–3569	775	60
SB11R	TATCTTGCATTGTGGATTTCCCTCATAA	4346–4319		
SB16F	ACGACGAAGACGAGGAGGTC	3752–3771	1,307	64
SB16R	AAAGTTGTGGCGTCTGGGGTT	5059–5039		
SB3F	GCTGCACGCGCAGTGGAAGTG	4729–4749	1,065	68
SB3R	TGCCTATCCTTTCGGAGTCGTTCC	5794–5771		

a *T_m_*, annealing temperature.

The CLRDV genome from Georgia characterized in this study was 5,868 bp long and encoded seven ORFs, as reported earlier for isolates from North and South America (8–10). It was 95 to 98% identical to the genome of other CLRDV isolates from the United States (Alabama, GenBank accession number MN071395; Texas, MN872302) and South America (KF359947, KF906261, KF906260, NC_014545, GU167940, and HQ827780) (Fig. 1C). The US isolates formed a clade separate from that of the South American isolates in the phylogenetic analysis based on nearly full-length nucleotide sequences (Fig. 1D).

### Data availability.

The nearly complete genome of CLRDV from Georgia described in this study was deposited in GenBank under accession number MT633122.
